# Small-sized polymeric micelles incorporating docetaxel suppress distant metastases in the clinically-relevant 4T1 mouse breast cancer model

**DOI:** 10.1186/1471-2407-14-329

**Published:** 2014-05-10

**Authors:** Yunfei Li, Mingji Jin, Shuai Shao, Wei Huang, Feifei Yang, Wei Chen, Shenghua Zhang, Guimin Xia, Zhonggao Gao

**Affiliations:** 1 State Key Laboratory of Bioactive Substance and Function of Natural Medicines, Institute of Materia Medica, Chinese Academy of Medical Science and Peking Union Medical College, 1 Xiannongtan Street, Beijing 100050, PR China; 2 Institute of Medicinal Biotechnology, Chinese Academy of Medical Science and Peking Union Medical College, Beijing 100050, PR China; 3 Pharmacy School, Yanbian University, Yanji 133000, PR China

**Keywords:** Small-sized polymeric micelles, Docetaxel, Metastasis, 4T1 Mammary carcinoma, Malignant breast cancer, Primary tumor resected

## Abstract

**Background:**

The small size of ultra-small nanoparticles makes them suitable for lymphatic delivery, and many recent studies have examined their role in anti-metastasis therapy. However, the anti-metastatic efficacy of small-sized nanocarriers loaded with taxanes such as docetaxel has not yet been investigated in malignant breast cancer.

**Methods:**

We encapsulated docetaxel using poly(*D,L*-lactide)_1300_-*b*-(polyethylene glycol-methoxy)_2000_ (mPEG_2000_-*b*-PDLLA_1300_) to construct polymeric micelles with a mean diameter of 16.76 nm (SPM). Patient-like 4T1/4T1^luc^ breast cancer models in Balb/c mice, with resected and unresected primary tumors, were used to compare the therapeutic efficacies of SPM and free docetaxel (Duopafei) against breast cancer metastasis using bioluminescent imaging, lung nodule examination, and histological examination.

**Result:**

SPM showed similar efficacy to Duopafei in terms of growth suppression of primary tumors, but greater chemotherapeutic efficacy against breast cancer metastasis. In addition, lung tissue inflammation was decreased in the SPM-treated group, while many tumor cells and neutrophils were found in the Duopafei-treated group.

**Conclusion:**

Small-sized mPEG_2000_-*b*-PDLLA_1300_ micelles could provide an enhanced method of docetaxel delivery in breast cancer metastasis, and may represent a valid chemotherapeutic strategy in breast cancer patients with resected primary tumors.

## Background

Metastasis is the main cause of breast cancer (BC)-related deaths [1]. Chemotherapy is currently used to prevent postoperative recurrence and metastasis and to prolong patient survival [2]. Nanotechnology has recently been acknowledged as a breakthrough in drug-delivery systems, and nanoparticles have emerged as promising carriers for anti-cancer drug delivery [3]. To date, over 20 nanoparticle therapeutics have been approved by the FDA for clinical use [4,5]. However, nanotechnology chemotherapy for cancer metastasis still presents a unique challenge, and has so far shown limited success. Increasing evidence suggests that the regulation of primary tumor growth differs from that of metastatic growth, and questions the clinical validity of using traditional, large-sized nanodrug systems. Doxil and Abraxane have provided only modest survival benefits because their large size hinders their delivery throughout the tumor [6-9], and single-use Doxil and Abraxane proved inefficient against BC metastasis [10,11]. Most current nano-therapeutic strategies focus on eliminating primary tumors based on enhanced penetration and retention (EPR) in well-vascularized primary tumors [12]. However, small metastases are usually poorly-vascularized and are not well-accessed by nanoparticles via the EPR effect, which is limited to tumors >4.8 mm in diameter, thus hindering the use of these nanoparticles for targeting small and poorly-vascularized metastases [13]. Generally, metastases present biological barriers because of their smaller size, higher dispersion to organs, and lower vascularization than primary tumors, making them less accessible to molecular and nanoparticle agents [14].

Malignant BC cells metastasize via the circulation system. Sentinel lymph nodes are typically the first site reached by disseminating malignant cancer cells, and are thus associated with an increased risk of distant metastasis and poor clinical outcome [15]. Although the primary tumor and affected lymph nodes can be removed by surgery, tumor cells remain inside the lymphatic vessels [16-18]. Preventing or inhibiting lymph node metastasis is thus critical for improving the outcome of BC patients, and the role of the lymphatic system as a major conduit for the proliferation and spread of pathologies, including metastatic BC [16-18], has directed attention on suitable drug-delivery strategies. Smaller nanodrugs (10–30 nm diameter) have recently proven effective against tumor metastasis and their mechanisms can be explained by lymphatic accumulation of these small-sized nanodrugs [19-22].

Transportation of agents by nanocarriers depends largely on agent structures [23], and these small-sized nanocarriers have been found to be suitable for incorporating water-soluble anthracyclines or platinum agents because of their electrostatic interactions and hydrophobic forces, but have not been shown to be suitable for hydrophobic taxanes (e.g. paclitaxel and docetaxel (DTX)) [19-22,24]. However, taxanes demonstrate a high level of clinical activity, represented by clinical remissions in advanced ovarian, breast and the upper aerodigestive tract cancers [25-27]. The central role of taxanes in the therapy of common epithelial cancers is further highlighted by their ability to induce remissions in patients with anthracycline- or *cis*-platinum-resistant epithelial cancers [27,28]. DTX, in particular, is broadly indicated for the treatment of non-small cell lung cancer, and breast, prostate, stomach and head and neck cancers [29], though these results remain open to debate [30], and is clinically preferred to paclitaxel [29]. However, to our best of our knowledge, very few studies have tested the efficacy of small-sized nanoparticles to deliver DTX in metastatic BC models, and the development of a small (10–30 nm) nanocarrier for DTX is desperately needed.

The safety of poly(*D,L*-lactide)-*b*-polyethylene glycol-methoxy(mPEG-*b*-PDLLA) mPEG-*b*-PDLLA-based polymeric micelles makes them one of the best delivery systems for small molecule anti-cancer drugs [31-34]. mPEG_2000_-*b*-PDLLA_1300_ in particular has been shown to be a good vehicle for taxanes, characterized by good physical stability [35,36]. In this study, we used mPEG_2000_-*b*-PDLLA_1300_ to prepare small-sized polymeric micelles encapsulating DTX (SPM), and subsequently evaluated their anti-metastatic efficiency in the highly-metastatic 4T1 mouse mammary tumor model. This tumor model recapitulates several features of advanced human BC, including the ability to generate spontaneous lung and lymph nodes metastases, and may be advocated as the model most closely representing the clinical situation in human cancer [37,38]. We compared the efficacy of SPM with free DTX (Duopafei) as a positive control. The therapeutic potential of SPM against metastasis was evaluated using bioluminescent imaging, lung tumor nodule examination, and histological examination.

## Methods

### Materials, cell line and animals

All reagents and solvents were used as received, without further purification. Monomethoxy polyethylene glycol with a molecular weight of 2000 Da (mPEG_2000_), *D,L*-lactide, and stannous octoate were purchased from Sigma-Aldrich Chemical Corp. (Shanghai, China); DTX was purchased from Beijing Norzer Pharmaceutical Co., Ltd. (China); free DTX (Duopafei) was manufactured by Qilu Pharm Co., Ltd. (Jinan, China); and 3-(4,5)-dimethylthiazol(-z-y1)-3,5-di-phenytetrazoliumromide (MTT) was obtained from Amresco (USA). Trypsin, fetal bovine serum (FBS) and RPMI-1640 medium were purchased from Hyclone (USA) and culture flasks and dishes were from Corning (USA). The 4T1 murine mammary adenocarcinoma cell line was kindly provided by Prof. Wei Liang of the Institute of Biophysics, Chinese Academy of Sciences. The 4T1^luc^ strain, which was genetically manipulated to overexpress the firefly luciferase gene, was previously engineered in our lab by Prof. Wei Huang. 4T1 and 4T1^luc^ cells were cultured in RPMI 1640 medium supplemented with 10% heat-inactivated FBS and incubated in a humidified atmosphere of 5% CO_2_ and 95% air at 37°C.

Female Balb/c mice (6–8-weeks old) were used for antitumor efficacy studies and were purchased from Beijing Vital River Laboratories (China). Animals were acclimatized in the holding facility prior to beginning the study. All animal procedures were approved by the Institutional Animal Care and Use Committee of the Chinese Academy of Medical Sciences. All surgeries were performed under sodium pentobarbital anesthesia (5 mg/mL solution), and all efforts were made to minimize suffering. Lung and liver sections were routinely stained with hematoxylin-eosin (HE) and evaluated under a light microscope.

### Synthesis of mPEG_2000_-*b*-PDLLA_1300_

mPEG_2000_-*b*-PDLLA_1300_ was synthesized by the ring-opening polymerization of *D,L*-lactide in the presence of mPEG_2000_ homopolymer and a catalyst, as described previously [35,36]. The molecular weights of copolymers were characterized by nuclear magnetic resonance (NMR) analysis using CDCl_3_ and a Mercury-400 spectrometer (Varian).

### Preparation and characterization of micelles encapsulating DTX

SPM was prepared by the conventional thin-film hydration method. Briefly, DTX (11.7 μmol) and mPEG_2000_-*b*-PDLLA_1300_ (15.2 μmol) were dissolved in acetonitrile (1 mL) in a round-bottomed flask to obtain a clear solution. The solvent was evaporated by rotary evaporation at 60°C for 1 h to obtain a solid DTX/copolymer matrix. Residual acetonitrile remaining in the film was removed under vacuum overnight at room temperature. The resultant thin film was hydrated with water at 60°C for 30 min to obtain a micelle solution.

SPM was extruded through a sterile membrane with pore size 220 nm (Millipore) to remove unincorporated DTX aggregates, and the sample was then diluted with distilled water to yield a final DTX concentration of 1 mg/mL, as determined by high-performance liquid chromatography (HPLC, Agilent 1200 series) with a pentafluorophenyl column (Curosil-PFP, 4.6 × 250 mm, 5 μm, Phenomenex, Guangzhou, China). The mobile phase was acetonitrile/water (50/50 (v/v)), and the flow rate was set at 1.0 mL/min. Size distribution was investigated by the dynamic light scattering (DLS) method using Nano ZS90 (Malvern Instruments Inc.). The morphology of SPM was characterized by transmission electron microscopy (TEM, H-7650, Hitachi, Japan).

Loading capacity was defined as the percentage of DTX by weight in the freeze-dried SPM, and encapsulation efficiency was defined as the percentage of DTX by weight incorporated in the micelles compared with the initial weight of DTX. After filtering through a membrane of pore size 220 nm, the SPM aqueous solution was freeze-dried (Epslon 1–4 LSC, Chirst, Germany). The loading amount of DTX was determined after dissolving freeze-dried SPM in acetonitrile (1:10, w/v) to completely destroy the core-shell structure of SPM. The SPM aqueous solution was diluted in acetonitrile (1:5, v/v) and the amount of DTX was then determined using a calibration curve established from standard solutions of DTX in acetonitrile by HPLC, using the above method. Each experiment was carried out in triplicate, and mean values ± standard deviations (SD) were calculated using the following formulae: Loading capacity (w/w%) = [(amount of physically loaded DTX)/(amount of SPM)] × 100%; Encapsulation efficiency (w/w%) = [(amount of physically loaded DTX)/(amount of DTX initially added)] × 100%.

The release profile of DTX from SPM was evaluated using a dialysis membrane method: 0.5 mL of SPM solution at a DTX concentration of 1 mg/mL was placed in a dialysis bag (molecular mass cut-off 3.5 kDa). The dialysis bag was incubated in 40 mL of phosphate-buffered saline (PBS, pH 7.4) at 37°C with gentle shaking at 100 rpm, and aliquots of incubation medium were removed at predetermined time points. DTX in the samples was quantified by HPLC using the above method.

### *In vitro* MTT cytotoxicity assay

The *in vitro* cytotoxicity was evaluated by MTT assay using the murine mammary cancer cell line 4T1^luc^. Briefly, cells were harvested from exponential-phase cultures, counted, and plated in 96-well flat-bottomed microtiter plates (5 × 10^3^/well). After 24 h incubation for adherence, cells were treated with a series of doses of Duopafei and SPM, respectively. After 48 h incubation, 20 μL of MTT (5 mg/mL) was added to each well of the plate followed by incubation for an additional 4 h. The MTT was then aspirated off and 200 μL/well of dimethyl sulfoxide was added to dissolve the formazan crystals. Finally, the optical density was measured at 490 nm using a microplate reader (Synergy H_1_/H_1_ MF, Bio-Tek Inc.). Untreated cells were used as control cells with 100% viability, and wells without MTT were used as blanks to calibrate the spectrophotometer to zero absorbance. The results were expressed as mean values ± SD of five measurements. The cell inhibition rate was calculated according to the formula: Inhibition rate (%) = [1-(OD_sample_-OD_blank_)/(OD_control_-OD_blank_)] × 100%.

### Cellular uptake of coumarin 6(C6)-loaded SPM (C6-SPM)

For *in vitro* fluorescence imaging, the near-infrared fluorescent probe C6 was loaded into mPEG_2000_-*b*-PDLLA_1300_ micelles to yield C6-SPM. Briefly, the polymers and excess C6 were co-dissolved in CHCl_3_ and a thin film was formed by the evaporation of CHCl_3_. PBS (pH 7.4) was added, followed by vortexing for 10 min. The micelle suspension was extruded through a sterile membrane of pore size 220 nm (Millipore) to remove free C6.

4T1^luc^ cells in exponential-stage growth were incubated with C6-SPM at 37°C for 5, 10, 20, and 30 min, respectively, rinsed three times with cold PBS, and then fixed with 4% paraformaldehyde for 10 min. Finally, cells were observed by confocal laser scanning microscopy (CLSM, TCS SP2, Leica, Germany). Images were examined using differential interference contrast and C6-SPM was recorded with the green channel (C6) with excitation at 488 nm.

### Cell apoptosis assay

Apoptotic cells were determined by dual staining with an Annexin V and propidium iodide (PI) kit (China KeyGEN Biosciences Company, China) according to the manufacturer’s instructions. After 48 h of culture in the exponential stage, 4T1^luc^ cells seeded in 12-well plates were treated for a further 48 h with 10 nmol/mL Duopafei or SPM, respectively. After treatment, cells were washed twice with warm PBS, detached by trypsin without EDTA, collected, centrifuged, washed with warm PBS, resuspended in the binding buffer and further stained with PI and Annexin V-FITC for 15 min at ambient temperature in the dark. Apoptosis was then analyzed using a FACScan cytometer equipped with Cell Quest software (BD Biosciences, USA). Quadrant analysis was performed and cells that stained positive for both Annexin V-FITC and PI were designated as apoptotic, while unstained cells were designated as live.

### Chemotherapy in unresected advanced 4T1^luc^ breast carcinoma model

A cell suspension (0.1 mL) containing approximately 4 × 10^5^ 4T1^luc^ cells at exponential stage was orthotopically injected into a mammary gland in the lower right quadrant of the abdomen of Balb/c female mice. Treatment commenced when the primary tumor diameter reached about 5–8 mm (day 9 after inoculation). The mice were divided randomly into three equal groups (n = 10/group) for treatment: negative control (5% glucose solution), Duopafei^,^ and SPM groups. Each treated group was injected intravenously via the tail vein at a dose of 10 mg DTX/kg body weight every 6 days for 24 days. Tumor volume (mm^3^) and bodyweights were measured simultaneously. The experiment was terminated at day 33 after inoculation.

### Chemotherapy in unresected advanced 4T1^luc^ breast carcinoma model using bioluminescent method

Cell suspensions (0.1 mL) containing approximately 4 × 10^5^ 4T1^luc^ cells at exponential stage were orthotopically injected into a mammary gland in the lower right quadrant of the abdomen of Balb/c female mice. Treatment commenced when the primary tumor diameter reached about 6–8 mm (day 9 after inoculation). The mice were divided randomly into two equal groups (n = 3/group) for treatment: Duopafei and SPM groups. No negative control group was established because the untreated mice were very weak and unable to tolerate multiple isoflurane anesthesia doses as a result of heavy lung tumor burdens. Each treated group was injected intravenously via the tail vein at a dose of 10 mg DTX/kg bodyweight every 6 days for 18 days. Anti-metastatic activity was evaluated by bioluminescence imaging as described above on days 9, 22, and 38 after inoculation. In brief, mice were administered the substrate *D*-luciferin (150 mg/kg in PBS) by intraperitoneal injection. Bioluminescence imaging was initiated 10 min after the injection. Mice received continuous exposure to 1–2% isoflurane to sustain sedation during imaging (IVIS-live Imaging System 200, Xenogen, PerkinElmer, USA). The acquisition time was the same for all image collections (30 s) and identical illumination settings were used for acquiring all images. The experiment was terminated at day 38 after inoculation. All mice were sacrificed and the lungs were harvested, fixed in Bouin’s solution for 24 h, and then photographed.

### Chemotherapy in unresected early-staged 4T1 breast carcinoma model

Approximately 1 × 10^4^ 4T1 freshly prepared tumor cells in exponential stage growth were injected into the lower right quadrant of the abdomen of BALB/c female mice. Treatment commenced when the primary tumor diameter reached around 1–2 mm (day 14 after inoculation). The mice were divided randomly into two equal groups (n = 6/group) for treatment: Duopafei and SPM groups. Each treated group was injected intravenously via the tail vein at a dose of 10 mg DTX/kg body weight every 3 days for 12 days. The experiment was terminated at day 26 after inoculation and all mice were sacrificed. Lungs and livers were harvested and fixed in 4% formalin solution for 72 h. The anti-metastatic efficacies of the treatments were evaluated by HE staining of paraffin-embedded tissues for histological examination of lungs and livers. Stained sections were examined and photographed.

### Postoperative chemotherapy in syngeneic murine 4T1^luc^ breast carcinoma model using bioluminescent method

Cell suspensions (0.1 mL) containing approximately 4 × 10^5^ 4T1^luc^ cells in exponential-stage growth were injected orthotopically into a mammary gland in the lower right quadrant of the abdomen of BALB/c female mice. The primary tumor was surgically removed when its diameter reached about 6–8 mm (day 20 after inoculation), as described previously [37,39]. Treatment commenced 7 days after surgery (day 27 after inoculation). The mice were divided randomly into three equal groups (n = 4/group) for treatment: negative control (5% glucose solution), Duopafei and SPM groups. Each treated group was injected intravenously via the tail vein at a dose of 5 mg DTX/kg body weight every 7 days for 21 days, and mouse bodyweights were measured simultaneously. Anti-metastatic activity was evaluated by bioluminescence imaging at day 48 after inoculation, as described above. Experiments were terminated at day 48 after inoculation. All mice were sacrificed and the lungs were harvested, fixed in Bouin’s solution for 24 h, and then photographed. The lungs were then washed in PBS and put into 4% formalin solution for 72 h, dehydrated, and embedded in paraffin for standard histological HE staining and photographing.

### Statistical analysis

Data were described as means ± SD of the indicated number of individual experiments. If there was significant variation between treatment and control groups, the mean values were compared using unpaired Student’s *t*-tests to assess the statistical significance of the differences. A *P* value of less than 0.05 was considered statistically significant, and a *P* value of less than 0.01 was considered highly statistically significant.

## Results

### Preparation and characterization of micelles

**Figure 1:**
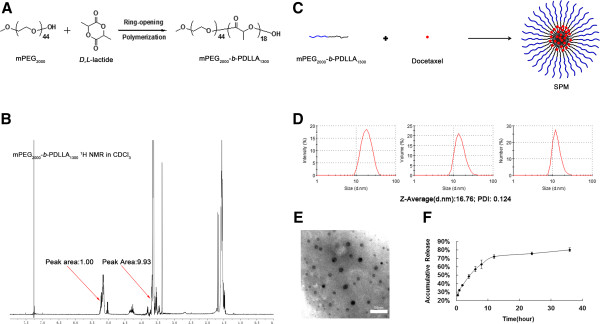
**Preparation and characterization of SPM. (A)** Synthesis scheme for mPEG_2000_-*b*-PDLLA_1300._**(B) **^1^H NMR spectra of mPEG_2000_-*b*-PDLLA_1300._**(C)** Schematic illustration of SPM. **(D)** Size distribution of SPM in aqueous medium measured by DLS analysis. **(E)** TEM images of SPM. (Scale bar: 50 nm). **(F)** *In vitro* release profile of SPM in PBS (pH 7.4).

mPEG_2000_-*b*-PDLLA_1300_ was successfully synthesized according to the scheme in Figure 1A. The degree of polymerization of PDLLA was calculated by comparing the integral intensity of the characteristic resonance of PDLLA at 5.2 ppm (-C(O)–C*H*(-CH_3_–)) and PEG resonance at 3.64 ppm (-OC*H*_2_C*H*_2_–) in the ^1^H NMR spectrum (as shown in Figure 1B). The calculated result indicated a mean molecular weight for mPEG-*b*-PDLLA of 3300 Da.

DTX was incorporated into SPM using the self-assembly procedure (Figure 1C). The DTX-loading and efficiency into the micelles were 15.57 ± 0.73% and 97.7 ± 1.03%, respectively. The sizes were examined by DLS. As shown in Figure 1D, SPM had a unimodal size distribution with a mean diameter of 16.76 nm, which was within the small-sized diameter range. TEM images showed that SPM was monodisperse and spherical and the TEM average size was determined as 13.77 nm (Figure 1E). The *in vitro* release behavior of SPM presented as the cumulative percentage release is shown in Figure 1F. The aforementioned polymer-metal complex formation between DACHPt and the carboxylic group of the P(Glu) in the PEG-*b*-P(Glu) led to the formation of polymeric micelles with the average diameters of approximately 30 nm [21], while doxorubicin and vinorelbine can be tightly packaged into small-sized PEG-PE-based micelles due to the amphiphilic nature of the drugs and PEG-PE molecules and their specific structures [20]. The ability of a micelle system to function as a solubilizer or a true carrier depends on its stability and the diversity of polymer chemistry can provide a custom fit to drug molecules to be loaded [23]. mPEG_2000_-*b*-PDLLA_1300_ was characterized as a good, small-sized carrier for DTX (Figure 1), given both the strong Van der Waals forces between the drug and the inner core of the micelles and the intermolecular H-bond between the hydroxyl and amide groups of DTX with the oxygen atoms in the PDLLA_1300_ chain, which were proposed to contribute to the structural stability of the micelles.

### *In vitro* cytotoxicity assays

We determined if encapsulation of DTX in SPM would increase drug entry into tumor cells and cytotoxicity *in vitro*. No blank micelle group was used in the MTT study because the amphiphilic copolymer mPEG-*b*-PDLLA has shown promising safety as a drug-delivery material in many FDA-approved clinical trials [31,33,34]. 4T1^luc^ cells were exposed to a series of equivalent concentrations of Duopafei and SPM for 48 h, and the inhibition rates were quantified using the MTT method.

**Figure 2:**
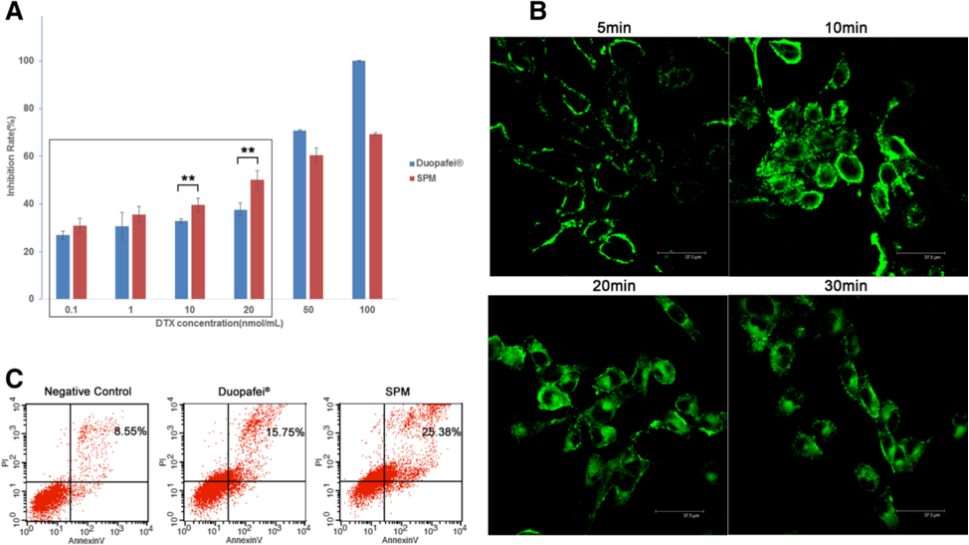
***In vitro *characterization of SPM. (A)** Cytotoxic effects of Duopafei and SPM in 4T1^luc^ cells, assessed by MTT assay. **P < 0.05. **(B)** CLSM images of 4T1^luc^ cells incubated with SPM for 5, 10, 20, and 30 min, respectively. (Scale bar: 37.5 μm). **(C)** Flow cytometry detected cell apoptosis in 4T1^luc^ cells incubated for 48 h with 10 nmol/mL Duopafei and SPM, respectively.

Tumor cell viabilities after 48 h incubation as a function of the amount of DTX in Duopafei and SPM demonstrated striking dose-dependent cytotoxicities (Figure 2A). mPEG_2000_-*b*-PDLLA_1300_ encapsulation accelerated cellular uptake of the drug and induced higher cytotoxicity in cancer cells, especially at lower DTX concentrations (0.1–20 nmol/mL). At DTX concentrations of 10–20 nmol/mL, the cytotoxicity of SPM was highly significantly greater than that of Duopafei (*P* < 0.01), indicating favorable *in vivo* drug delivery. However, 4T1^luc^ cells were more sensitive to Duopafei than SPM at higher concentrations (50–100 nmol/mL). Micelles are internalized into cancer cells via endocytic mechanisms [40], while the free drug diffuses into cells according to the concentration gradient between the intracellular and extracellular environments, explaining why Duopafei is more cytotoxic at higher concentrations. These results indicate that encapsulation of DTX in mPEG_2000_-*b*-PDLLA_1300_ may significantly enhance the drug’s cytotoxicity.

### *In vitro* cellular uptake of SPM

Cellular uptake plays a key role in the nanodrug delivery system. Poor cellular uptake may result in low levels of intracellular DTX, ultimately leading to unsatisfactory therapeutic effects. Cellular uptake of C6-SPM was qualitatively visualized by CLSM and the internalization speed was roughly estimated. The CLSM images of 4T1^luc^ cells after incubation with C6-SPM for 5, 10, 20, and 30 min are shown in Figure 2B. CLSM images at 5 and 10 min showed that C6-SPM fluorescence (green) was closely located around the membrane, indicating that C6-SPM had not been internalized into 4T1^luc^ cells. However, when the incubation time was extended to 20 min, C6-SPM was successfully internalized into 4T1^luc^ cells. Micelles have previously been reported to be internalized into the cytoplasm together with the entrapped drug via an endocytic mechanism [40], which process is demonstrated in Figure 2B.

### SPM increased DTX-induced apoptosis in 4T1^luc^ cells

DTX has beenwas described as anthe antimitotic agent that binds to β-tubulin, resulting in block of the cell cycle block at the G_2_/M phase and apoptosis of cells [27,29]. E, enncapsulation of DTX in nanoparticles could induce moreincreased apoptosis inof prostate cancer cells through the activation of the caspase-2 pathway [41].Given that SPM demonstrated stronger *in vitro* cytotoxicity than free DTX, we performed apoptosis assays using Annexin V-FITC and PI staining to compare apoptosis induction by SPM and Duopafei. As predicted, SPM increased late apoptosis in 4T1^luc^ cells compared with Duopafei (25.38% vs 15.75%) (Figure 2C).

### Primary tumor inhibition by SPM and Duopafei *in vivo*

**Figure 3:**
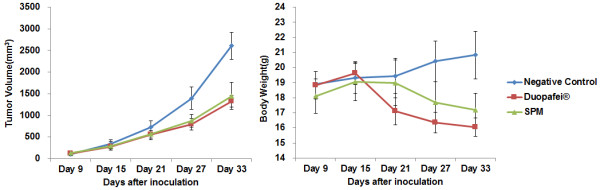
**Primary tumor-volume and bodyweight changes in Duopafei- and SPM-treated mice bearing 4T1**^**luc **^**tumors.**

Nanoparticles can spontaneously extravasate and accumulate in the tumor interstitium by the EPR effect as a result of the unique fenestrated vascular architecture and poor lymphatic drainage [3,13]. We investigated if incorporation of DTX into micelles could improve their efficacy against primary tumors in the same way. The mean primary tumor volumes on day 33 after treatment with Duopafei or SPM were 1300–1400 mm^3^, compared with up to 2600 mm^3^ in the negative control group (Figure 3). However, tumor growth in Duopafei-treated mice was slower than in SPM-treated mice, though the difference was not significant. All 4T1 tumors, regardless of their size, are highly vascularized [37]. These results suggest that SPM did not exert an EPR advantage against primary tumors compared with Duopafei. Concerning the bodyweight curve, SPM-treated mice lost less bodyweight than Duopafei-treated mice, but the difference was not significant, possible because of spontaneous damage to the body associated with the development of metastases. SPM thus failed to demonstrate significant superiority to Duopafei in terms of suppressing primary tumor growth.

### SPM prevents lung metastasis in advanced unresected BC animal model

**Figure 4:**
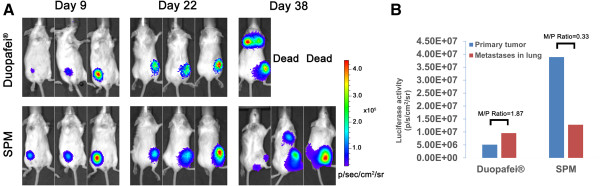
**Effects of SPM and Duopafei on cancer cell dissemination following primary 4T1**^**luc **^**tumor resection. (A)** *In vivo* bioluminescent images of Duopafei- or SPM-treated mice bearing 4T1^luc^ tumors. Two mice in the Duopafei-treated group had died of metastases by day 38 after inoculation. **(B)** Primary tumor and lung metastasis progression in the Duopafei- and SPM-treated groups were quantitatively monitored at day 38 after inoculation using biophotonic imaging analysis. M/P Ratio represents the ratio of luciferase activity in lung metastases to that in the primary tumor.

**Figure 5:**
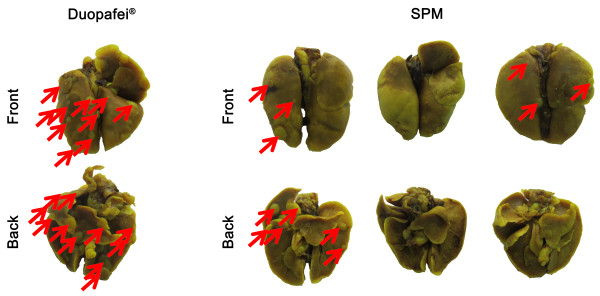
**Lung histology in the Duopafei- and SPM- treated groups.** Lungs were immersed in Bouin’s fixative for 24 h. Red arrow indicates the metastatic nodule on the lung.

4T1^luc^ cells express luciferase to visualize the metastasis foci. Bioluminescence imaging can therefore be used to provide a simultaneous and sensitive analysis of multiple tissues/organs and to evaluate the anti-metastatic efficacies of agents [42,43]. We compared the anti-metastatic abilities of SPM and Duopafei in unresected BC in 4T1^luc^ mice using bioluminescence imaging and metastatic nodule examination (Figures 4 and 5). The results of longitudinal imaging of the thoracic region and lower limbs at 9, 22 and 38 days after inoculation are shown in Figure 4. At Day 38, the primary tumors generally reached ≥1 cm^3^ and metastasis to the thoracic region was apparent in some mice. The relative level of bioluminescence correlates with the cancer burden [43]. As demonstrated in Figure 4A, two mice were dead at day 38 after inoculation and only one mouse survived in the Duopafei-treated group, but heavy lung metastasis was indicated by strong luciferase activity in the thoracic region. In contrast, all the mice in the SPM-treated group survived and only one showed strong luciferase activity in the thoracic region.

To validate the bioluminescence results in Figure 4A, lung foci were apparent as white nodules following immersion of the lungs in Bouin’s fixative for 24 h. The number of tumor nodules was taken as an indicator of varying metastasis in a previous study of tumor cell colonization in the lungs [20]. However, it cannot reflect the metastatic situation in the lung adequately because of differences in tumor nodule sizes; i.e., lungs with more but smaller nodules do not necessarily indicate more advanced metastasis than lungs with fewer but larger metastatic nodules. We therefore used photographs to reflect lung metastasis development more accurately. As shown in Figure 5, many large metastatic nodules were found in one mouse in the Duopafei-treated group and one mouse in the SPM-treated group, resulting in strong luciferase activity (Figure 4A). However, one mouse lung had three small metastatic nodules (indicated by red arrows in Figure 5), which differed slightly from the bioluminescence imaging result in Figure 4A. This discrepancy may be associated with limitations of the bioluminescence method, given that the fewer photons generated by smaller metastatic lesions are less easily detected by the device. Bioluminescence imaging was performed using the Xenogen *In Vivo* Imaging System, which consists of a supersensitive cooled charge-coupled device camera mounted inside a light-tight imaging chamber. However, the camera is only capable of detecting a minimum radiance of 100 photons per second per square centimeter per steridian [44], and the luciferase signals of small metastatic foci usually disappear when imaged together with large primary tumors with much stronger luciferase activity.

Lung metastasis surveillance was performed both visually (Figure 4A) and quantitatively by bioluminescence (Figure 4B). As shown in Figure 4B, although there were fewer absolute metastatic tumor cells in the Duopafei-treated mouse compared with SPM-treated mice, the ratio between the primary tumor and lung metastases was much higher in the Duopafei- than in the SPM-treated group. The 4T1^luc^ primary tumor has been shown to play an important role in promoting metastatic proliferation [39], and these results suggest that SPM could suppress metastatic proliferation from the primary tumor more efficiently than Duopafei.

### SPM suppresses lung and liver metastases in early-stage unresected BC animal model

**Figure 6:**
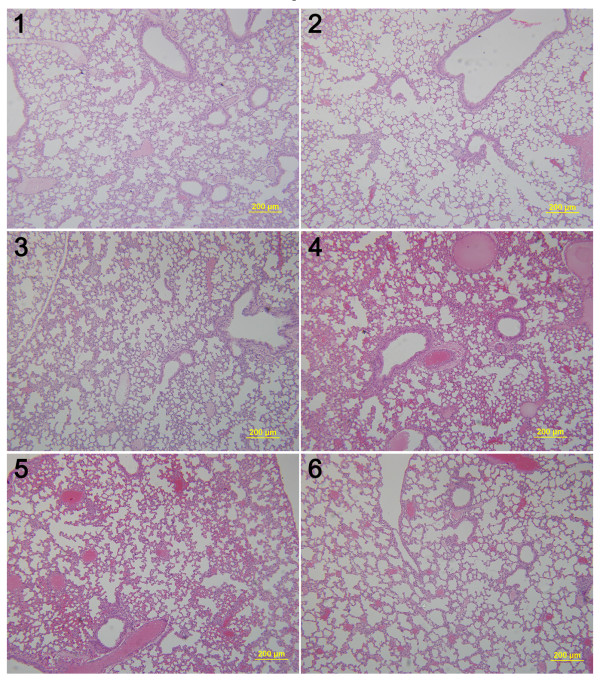
**Representative histopathological images of lungs from Duopafei-treated mice. 1**, **2**, **3**, **4**, **5** and **6** indicate individual mice (n = 6) bearing 4Tl primary tumors with a diameter of 1–2 mm. (Scale bar: 200 μm).

**Figure 7:**
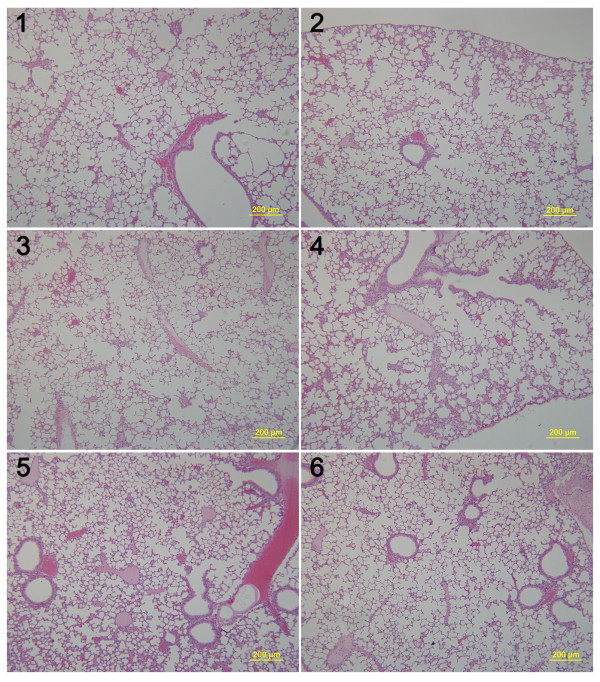
**Representative histopathological images of lungs from SPM-treated mice. 1**, **2**, **3**, **4**, **5** and **6** indicate individual mice (n = 6) bearing 4Tl primary tumors with a diameter of 1–2 mm. (Scale bar: 200 μm).

**Figure 8:**
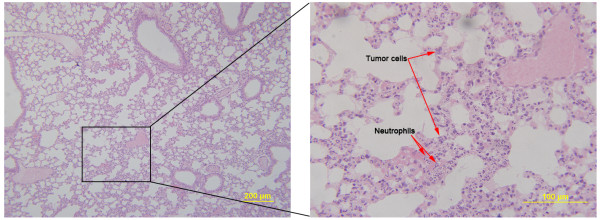
**Representative lung image from Duopafei-treated mouse.** HE-stained lung from mouse no.1 showing metastatic cancer cells adjacent to blood vessels. Many identifiable neutrophils located extravascularly were also observed in the lung tissue. (Left scale bar: 200 μm; right scale bar: 100 μm).

**Figure 9:**
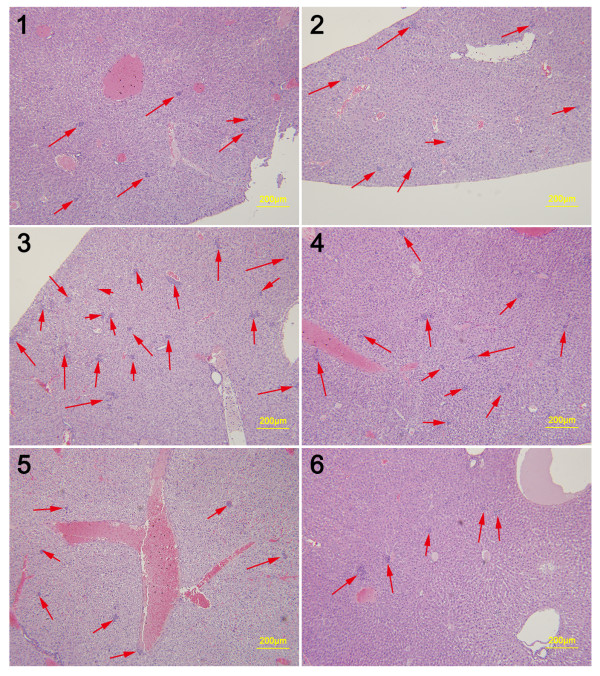
**Representative liver images from Duopafei-treated mice. 1**, **2**, **3**, **4**, **5** and **6** indicate individual mice (n = 6) bearing 4Tl primary tumors with a diameter of 1–2 mm. Red arrows indicate metastatic foci in the liver. (Scale bar: 200 μm).

**Figure 10:**
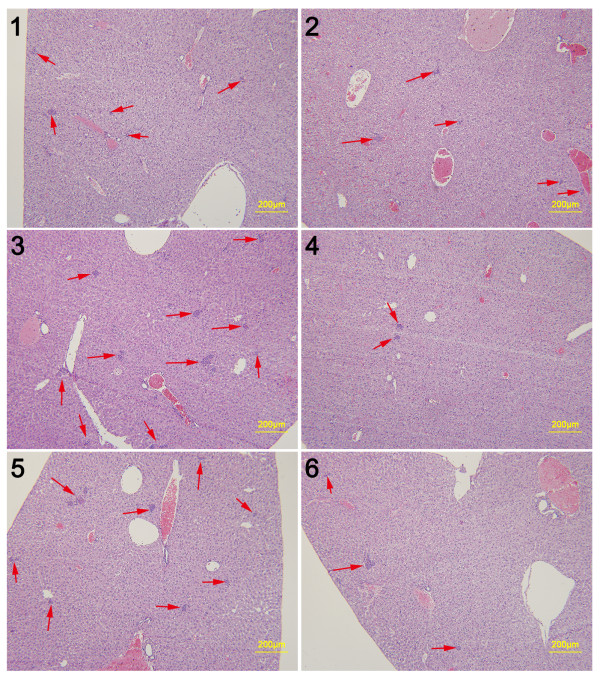
**Representative liver images from SPM-treated mice. 1**, **2**, **3**, **4**, **5** and **6** indicate individual mice (n = 6) bearing 4Tl primary tumors with a diameter of 1–2 mm. Red arrows indicate metastatic foci in the liver. (Scale bar: 200 μm).

SPM was shown above to decrease the formation of lung metastases in mice with advanced BC. 4T1 primary tumors >3–4 mm in diameter that have been in place for 2 weeks are similar to advanced human BC [37]. We also assessed the ability of SPM to reduce metastasis in early-stage malignant BC. We inoculated as few as 1 × 10^4^ 4T1 cells into the mammary fat pad of Balb/c mice, sacrificed all mice when the primary tumors reached 1–2 mm in diameter, and then evaluated metastasis development in the lung and liver by HE staining (Figures 6 and 7). Given that inflammatory processes are known to be involved in metastasis [17,42], we evaluated leukocyte infiltration in the lung, as well as tumor cell metastasis. Lung tissue from SPM-treated mice showed barely measurable levels of tumor cells (Figure 7) and few neutrophils infiltrated by tumor cells. In contrast, many tumor cells and neutrophils were found in close proximity to vessels (Figures 6 and 8). The protumoral effect of chronic inflammation has been extensively studied. The tumor microenvironment contains many resident cell types, such as adipocytes and fibroblasts, but is also populated by migratory hematopoietic cells, most notably macrophages, neutrophils and mast cells, which play pivotal roles in the progression and metastasis of tumors [17,42,45]. Fewer leukocytes, especially neutrophils, may have had an indirectly favorable effect on lung metastasis in the SPM-treated group. We also compared the HE results of liver metastases (Figures 9 and 10). Extensive metastases were found in the livers of all mice, but mice treated with SPM showed fewer liver metastases than those treated with Duopafei. Overall, these results suggest that SPM could suppress metastases in distant organs such as the lungs and liver in early-stage malignant BC.

### SPM suppresses metastasis in a postoperative chemotherapy BC animal model

To simulate the real clinical situation, we examined the anti-metastatic efficacy of SPM in a postoperative model of the 4T1^luc^ mammary tumor. Mice were inoculated orthotopically with 4T1^luc^ cells in the fourth mammary pad and the primary tumors were allowed to grow progressively, become extensively vascularized, and to metastasize. The primary tumors were then surgically resected when their sizes reached 6–8 mm (day 20 after inoculation) and chemotherapy was initiated (Additional file 1). To ensure the recovery of all mice, chemotherapy began from the seventh day after surgery (day 27 after inoculation).

**Figure 11:**
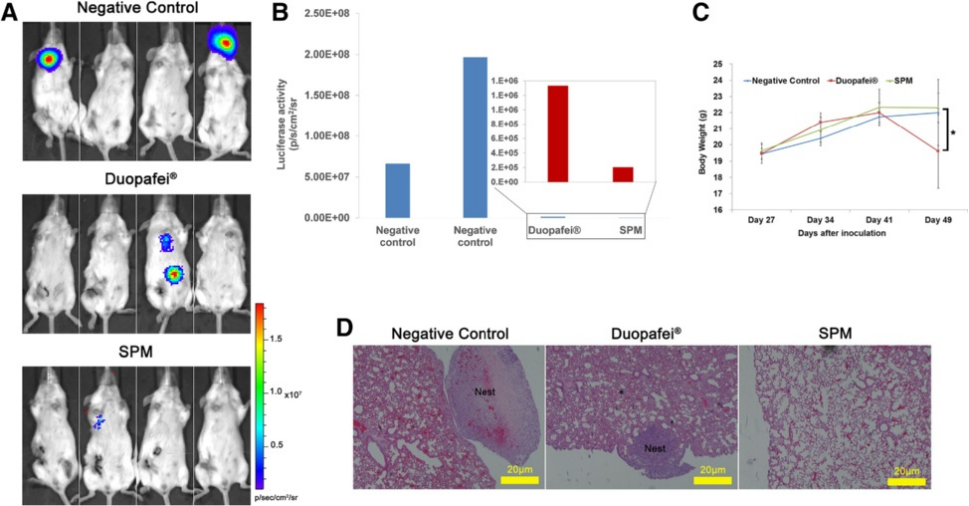
**Effects of SPM and Duopafei on metastasis burden in resected 4T1**^**luc **^**chemotherapy animal model. (A)** *In vivo* bioluminescent images of mice (n = 4) after treatment with 5% glucose solution (negative control), Duopafei, or SPM. **(B)** Metastases progression was monitored quantitatively in the Duopafei- and SPM-treated groups at day 49 after inoculation using biophotonic imaging analysis. **(C)** Bodyweight changes in mice treated with 5% glucose solution (negative control), Duopafei, and SPM. Significant bodyweight loss was observed in the Duopafei-treated group compared with the SPM-treated group (*P* < 0.05). **(D)** Representative histopathological images of lungs in the 5% glucose solution (negative control), Duopafei- and SPM-treated groups. (Scale bar: 20 μm).

**Figure 12:**
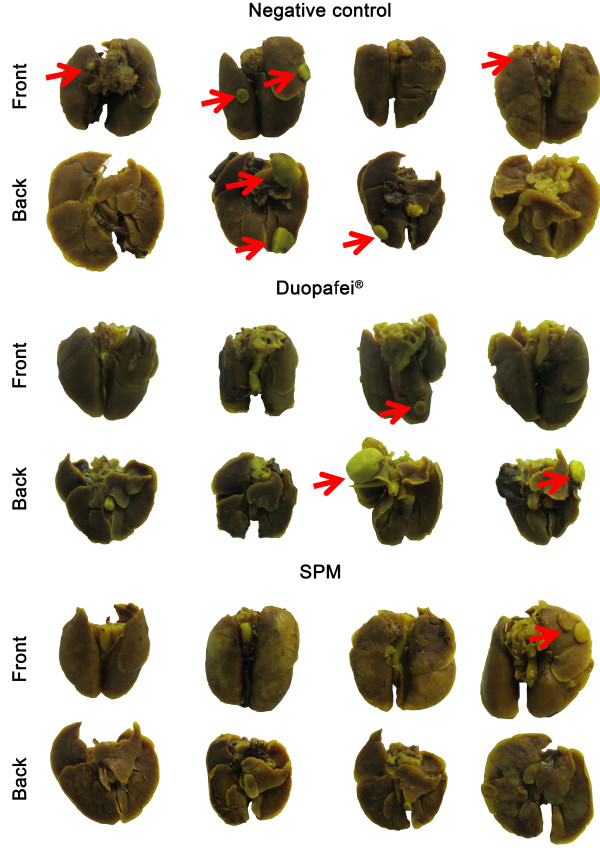
**Histological images of lungs in Duopafei- and SPM-treated mice.** Lungs were examined after immersion in Bouin’s fixative for 24 h in mice treated with 5% glucose solution (negative control), Duopafei or SPM. Red arrows indicatet metastatic foci on the lung.

Bioluminescent images of mice treated with 5% glucose solution (negative control) or different DTX formulations are shown in Figure 11A. By day 48 after inoculation, SPM-treated mice showed better metastasis control with less luciferase activity compared with Duopafei, which showed modest anti-metastatic effects (Figure 11B). In addition to bioluminescence imaging, lung metastatic nodules were photographed and examined following immersion in Bouin’s fixative for 24 h. Two small metastatic nodules were found in the lung of one mouse in the SPM-treated group, while larger tumor nodules were found in two mice in the Duopafei-treated group, indicating more severe metastasis in the latter group (Figure 12). The results of nodule examination differed slightly from the bioluminescence imaging results for the reasons discussed above. However, metastases in mice treated with SPM were dramatically decreased, whereas free DTX treatment only slightly reduced metastasis development. Surgical removal of the primary tumors has the advantage of reflecting the clinical situation where the primary breast tumor is surgically removed and the metastatic foci remain intact [46,47], and these results therefore provide valuable information for clinical application.

Compared with the negative control group, SPM-treated mice showed no bodyweight loss while Duopafei-treated mice showed significant bodyweight loss (*P* < 0.05) on day 49 after inoculation (Figure 11C). There were no significant changes in gross measurements such as skin ulceration, toxic death, behavior, or feeding in the SPM-treated group. It is possible that SPM’s stability prevented random DTX release into the body, while its small size enhanced its anti-metastatic efficacy.

As reported in previous studies [17,42,45] and as discussed above, inflammation is believed to be associated with cancer metastasis. In the present study, histological evidence (Figure 11D) showed concomitant anti-inflammatory activity in the lungs of SPM-treated mice in accordance with Figures 6, 7 and 8. Whole-lung HE images in mice with/without primary tumor resection indicated that SPM could control metastasis development more efficiently than free DTX.

## Discussion

Syngeneic murine models of metastatic BC are imperative for testing new therapeutics in a preclinical setting. Highly-malignant 4T1 mammary carcinoma cells, originally isolated by Miller and colleagues, provides the most popular spontaneous BC model [1,46]. In contrast to other experimental animal models in which tumor growth and progression do not parallel their human counterparts, many characteristics of 4T1 tumors in syngeneic mice resemble those of human mammary carcinomas, making the 4T1 model suitable for studies of metastatic cancer [1,42,48,49]. Moreover, 4T1 tumors are deemed to be one of the best transplantable BC models in current use [38].

In this study, we prepared SPM, an mPEG_2000_-*b*-PDLLA_1300_-based DTX polymeric micelle formulation with a particle size (i.e., 10–30 nm) previously shown to result in maximal accumulation in lymph nodes [20,21]. Compared with free DTX (Duopafei), SPM was more effective in inhibiting the growth of 4T1 tumor cells *in vitro* by inducing late apoptosis. In animal models with unresected primary tumors resected, SPM at a safe dosage (10 mg DTX/kg body weight) remarkably inhibited BC metastasis to distant major organs such as the lungs and liver compared with Duopafei.

In most clinical situations, primary mammary tumors are treated by surgery, but approximately 33% of women successfully treated for primary tumors still die subsequently from spontaneous metastatic disease [50]. Primary 4T1 tumors can readily be removed surgically, allowing metastatic disease to be studied in an animal setting comparable to the clinical situation [37]. In animal models with resected primary tumors, SPM demonstrated greater inhibition of 4T1^luc^ cell dissemination and fewer signs of toxicity compared with Duopafei. Previous studies have reported differences in the effectiveness and toxicities of micelle-encapsulated drugs [19-24], indicating that the choice of nanocarrier is critical, and that cancer therapies based on polymeric micelles can improve drug efficacy without increasing the drug dose, and while also reducing systemic toxicity. The present results validated SPM as an effective future treatment for preventing the metastasis of malignant BC.

The lymph nodes are typically the first site reached by disseminating malignant BC cells, correlated with an increased risk of distant metastasis and poor clinical outcome [15]. Although the primary tumor and affected lymph nodes can be removed during surgery, tumor cells remain inside the lymphatic vessels [16-18]. SPM did not outperform Duopafei in terms of suppressing growth of the primary tumor [39], but dramatically suppressed metastasis, especially in mice with resected primary tumors. The enhanced anti-metastatic efficacy of SPM may be attributed to its lymphatic accumulation associated with its small size and increased delivery to the lymphatic system, which is consistent with previous observations using other kinds of anti-cancer drugs [19-22,24].

Many solid tumors, including breast tumors, are associated with local inflammation [51]. Indeed, cancer-related inflammation has recently been proposed as the seventh hallmark of cancer [52]. A previous study of 4T1 tumors observed a progressive increase in hematopoiesis throughout the 6-week time course as primary tumors progressed and metastases developed at distant sites [42]. This was further evidenced by increasing levels of circulating neutrophils and the development of inflammation in the lungs in the present study. The decreased inflammation in the lungs in the SPM-treated group confirmed that SPM slowed the development of metastases in the lung and other distant major organs.

The present study had some limitations. Despite considerable progress in demonstrating the anti-metastatic effects of SPM against malignant BC, the molecular basis underlying its inhibitory functions remains obscure and further experiments are needed to address this issue.

## Conclusions

In conclusion, SPM encapsulating DTX showed greater anti-metastatic efficacy than Duopafei in the clinically relevant BC spontaneously metastatic mouse model. To the best of our knowledge, this is the first study to investigate the use of DTX and mPEG_2000_-*b*-PDLLA_1300_ as a small-sized drug-delivery system for preventing the metastasis of malignant BC. The particle size of SPM is suitable for blocking the lymph node pathway during the cancer cell dissemination process. As an alternative to free DTX, SPM may show reduced toxicity and improved anti-metastatic efficacy. The results of this study indicate that SPM represents a promising clinical approach for treating metastatic disease associated with malignant BC.

## Abbreviations

DTX: Docetaxel; SPM: Small-sized polymeric micelles loaded with docetaxel; FBS: Fetal bovine serum; BC: Breast cancer; PBS: Phosphate-buffered saline (pH 7.4); EPR: Enhanced penetration and retention; HE: Hematoxylin and eosin; HPLC: High-performance liquid chromatography; DLS: Dynamic light scattering; MTT: 3-(4,5)-dimethylthiazol(-z-y1)-3,5-di-phenytetrazoliumromide; PI: Propidium iodide; C6: Coumarin 6; C6-SPM: Small-sized polymeric micelles loaded with coumarin 6; CLSM: Confocal laser scanning microscope; TEM: Transmission electron microscopy.

## Competing interests

The authors declare that they have no competing interests.

## Authors’ contributions

YFL contributed to the study concept and design, experiment execution, data collection and interpretation, statistical analysis, manuscript drafting and editing, and literature research. MJJ contributed to the literature research, experiment design and manuscript drafting and editing. SS contributed to nanoparticle preparation and characterization, animal experiment execution and data collection. WH contributed to the design of preliminary *in vitro* and *in vivo* experiments. FFY participated in execution of preliminary *in vitro* experiments. WC participated in execution of preliminary *in vivo* experiments. SHZ contributed to the execution of all the bioluminescent imaging experiments. GMX contributed to the literature research and manuscript drafting and editing. ZZG contributed to the study concept and design, manuscript drafting and editing, and data analysis and interpretation and is the corresponding author. All authors read and approved the final manuscript.

## Pre-publication history

The pre-publication history for this paper can be accessed here:

http://www.biomedcentral.com/1471-2407/14/329/prepub

## Supplementary Material

Additional file 1 Surgical removal of the primary tumor. Click here for file
