# Supplementary figures and images for: Small-sized polymeric micelles incorporating docetaxel suppress distant metastases in the clinically-relevant 4T1 mouse breast cancer model
Source: BMC Cancer. 2014 May 10;14:329. doi: 10.1186/1471-2407-14-329 (PMC4023534; doi:10.1186/1471-2407-14-329)

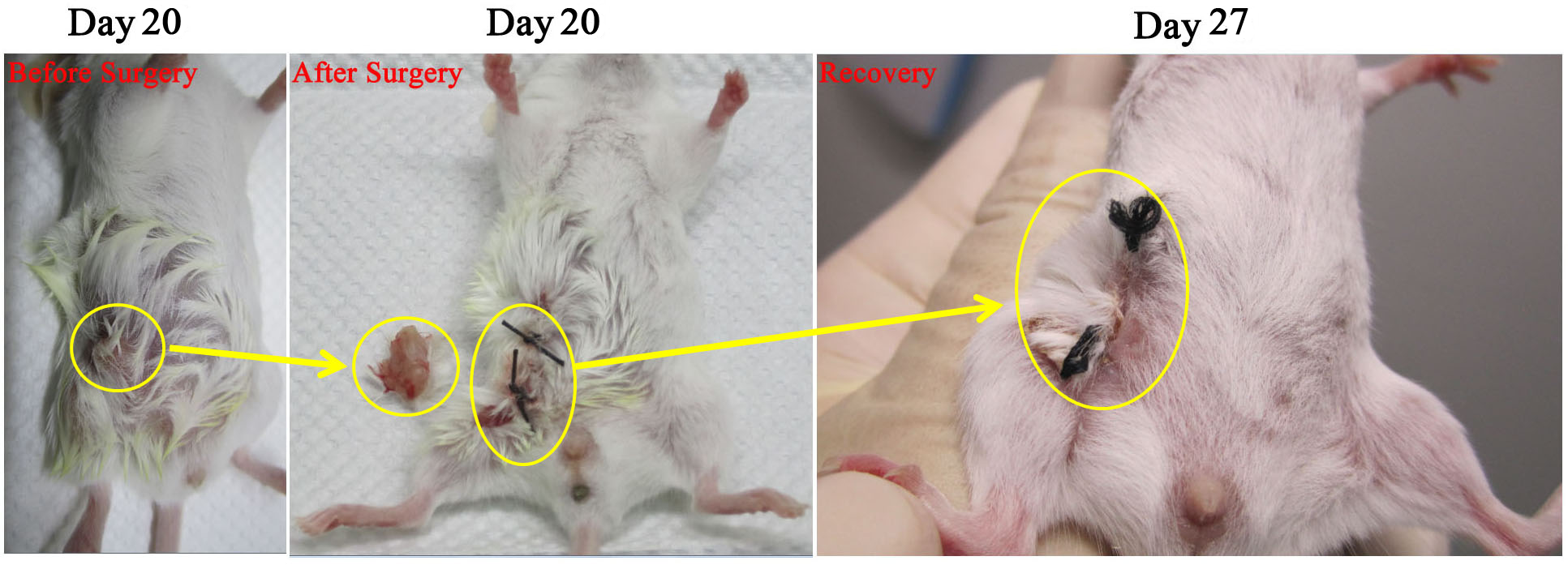

Supplement: Additional file 1 — Surgical removal of the primary tumor. [file 1471-2407-14-329-S1.tiff]
